# Metal Fluorides, Metal Chlorides and Halogenated Metal Oxides as Lewis Acidic Heterogeneous Catalysts. Providing Some Context for Nanostructured Metal Fluorides

**DOI:** 10.3390/molecules22020201

**Published:** 2017-01-28

**Authors:** David Lennon, John M. Winfield

**Affiliations:** School of Chemistry, University of Glasgow, G12 8QQ Glasgow, UK; David.Lennon@glasgow.ac.uk

**Keywords:** metal fluoride, metal fluoride, oxohalide, catalysis, lewis acid, oxychlorination, chlorofluorocarbon, chlorohydrocarbon

## Abstract

Aspects of the chemistry of selected metal fluorides, which are pertinent to their real or potential use as Lewis acidic, heterogeneous catalysts, are reviewed. Particular attention is paid to β-aluminum trifluoride, aluminum chlorofluoride and aluminas γ and η, whose surfaces become partially fluorinated or chlorinated, through pre-treatment with halogenating reagents or during a catalytic reaction. In these cases, direct comparisons with nanostructured metal fluorides are possible. In the second part of the review, attention is directed to iron(III) and copper(II) metal chlorides, whose Lewis acidity and potential redox function have had important catalytic implications in large-scale chlorohydrocarbons chemistry. Recent work, which highlights the complexity of reactions that can occur in the presence of supported copper(II) chloride as an oxychlorination catalyst, is featured. Although direct comparisons with nanostructured fluorides are not currently possible, the work could be relevant to possible future catalytic developments in nanostructured materials.

## 1. Introduction

Close-packed, solid metal fluorides have, for the most part, relatively small surface areas, although in some cases the surface metal cations have significant Lewis acidity due to their highly electronegative fluoride anion nearest-neighbors. For heterogeneous catalysis, partially fluorinated oxide surfaces have often been preferred, in view of their significantly increased surface areas without great loss in Lewis acidity.

The development of nanostructured metal fluorides, which has been rapid over the past 10–15 years [1], has resulted in renewed interest in the possible catalytic applications of these high-surface-area metal fluorides and it is timely to consider how they compare as catalysts with materials prepared by more conventional routes. Catalytic studies are described explicitly in other contributions to this themed collection; here, in the first part of the review, we describe some surface properties of what might be termed ‘competitor catalysts’. The emphasis is on various forms of aluminum fluoride and γ-alumina, which have been fluorinated using various fluorinating agents. Although fluorinated chromia is also an obvious comparator, its catalytic activity is not dealt with here, since we have very recently compiled a detailed account of this topic [2].

## 2. Aluminum Fluorides and Halogenated Aluminas

Two of the important aluminum fluorides, which can be regarded as precursors to nanoscale metal fluorides, are β-aluminum trifluoride and aluminum chlorofluoride (ACF). The former contains a six-coordinate Al^III^ but, unlike α-AlF_3_, which is close-packed, has the more open hexagonal tungsten bronze (HTB) structure [3]. This phase behaves as a solid Lewis acid and has significant catalytic behavior [4]; a model for the coordinatively unsaturated surface Al^III^ species has been deduced [5]. In contrast, ACF was developed from industrial research in fluorocarbon chemistry [6]. Judged by its chemical behavior, its Lewis acidity is comparable to that of liquid antimony pentafluoride, a benchmark in the field. Synthesized by halogen exchange between solid aluminum trichloride and various chlorofluorocarbons, hydrochlorofluorocarbons or hexafluoropropene, the reactions do not result in complete replacement of Cl by F; the stoichiometry of the solid product is AlCl*_x_*F_3−*x*_, with x being in the range 0.05–0.3. Surface areas are in the range 100–150 m^2^·g^−1^. The structure of the amorphous solid has been deduced at the atomic level from a number of spectroscopic techniques [7]. Chlorine is distributed throughout the solid, rather than in a discrete AlCl_3_ phase, and is possibly bridged to three Al^III^ centers. There appear to be three different types of six-coordinated Al^III^.

The defect spinel oxide γ-alumina is an ideal base material to investigate the effects of fluorination upon an oxide surface. The surface properties depend on both the reagent employed and the conditions used, as illustrated in Table 1.

**Figure 1:**
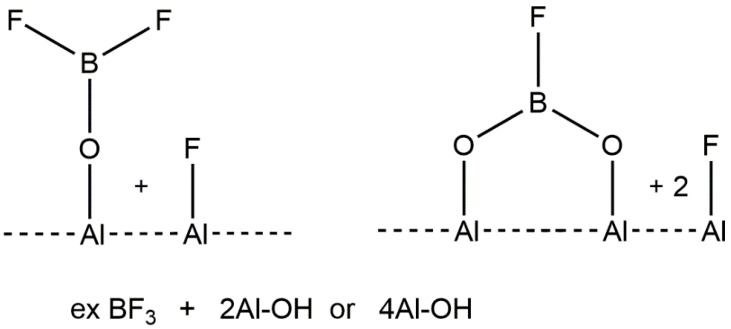
Possible surface species derived from BF_3_ and oxides. Redrawn from Reference [9].

These fluorinated aluminas are not supported reagents, since the fluorine is incorporated within the surface, and in some cases, possibly in the bulk to some extent. They contrast therefore with materials, such as supported boron trifluoride [9], which have been used widely as solid Lewis acids. Two of the possible surface species that have been suggested are shown in Figure 1.

The uptake of F by γ-alumina is slow when CHF_3_ is used. The process is initiated at the surface of oxide particles, subsequently being incorporated into sub-surface regions. The reaction between sulfur tetrafluoride and γ-alumina is essentially a hydrolysis; the course of the reaction has been followed using the radiotracers fluorine-18 and sulfur-35. The hydrolysis involves the replacement of surface hydroxyls by F and the partial replacement of bridging Al-O-Al moieties by Al-F, with the concomitant formation of OSF_2_ and SO_2_. These are not strongly adsorbed. The Lewis acid sites have disordered F/O environments rather than being fully fluorinated. When the SF_4_ treatment is performed under static conditions, nominally at room temperature, the retention of the co-product, HF, appears to be complete. Consequently, γ-alumina fluorinated by this method possesses Brønsted acidity in addition to the expected Lewis acidity.

A most powerful method to probe the Lewis acidity of fluorinated surfaces is FTIR spectroscopy using Lewis base probe molecules such as pyridine (py) or carbon monoxide, and 2-methyl-substituted py as a probe where Brønsted surface hydroxyl groups are present. Studies using CO at low temperatures can be particularly powerful, since different types of surface sites can be detected and compared. The aluminum fluorides mentioned above and various oxofluorides of different structural types have been studied in this way and the results compared with nanoscale aluminum fluorides; accounts of the methodology and some illustrative results are to be found in [10,11].

Useful data can be obtained in addition, using a radiolabeled probe molecule to investigate a fluorinated surface. Experiments using anhydrous hydrogen chloride and its precursors, labeled with chlorine-36, are described below. In addition, a radiotracer can provide important mechanistic details.

**Figure 2:**
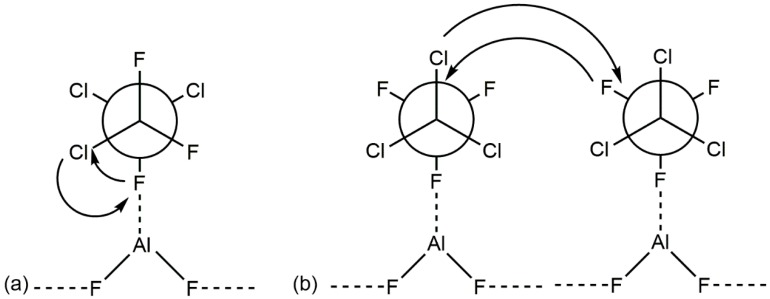
Schematic representation of possible adsorbed states for (**a**) CCl_2_FCClF_2_ prior to isomerization to CCl_3_CF_3_; and (**b**) CCl_3_CF_3_ prior to its dismutation. Redrawn from Reference [12].

Two examples are provided by the behavior of the trichlorotrifluoroethane isomers at β-AlF_3_ and the fluorinated γ-alumina surfaces. Isomerization of CCl_2_FCClF_2_ to the thermodynamically preferred isomer CCl_3_CF_3_ does not involve any formation of Al-Cl surface bonds, as indicated by the labeling of CCl_2_FCClF_2_ with [^36^Cl]. This indicates that the isomerization proceeds by an intra- rather than by an inter-molecular mechanism, as shown in Figure 2a. Isomerization is followed by a dismutation reaction giving a mixture of CCl_2_FCF_3_ and CCl_3_CClF_2_, which, from the lack of the incorporation of [^36^Cl] into the solid surface, is believed to involve a halogen exchange between two adsorbed CCl_3_CF_3_ molecules, as shown in Figure 2b [12].

These observations provide indirect evidence for the presence of strong Lewis acid surface sites on β-AlF_3_ and fluorinated γ-aluminas, sufficiently strong to allow C–F bonds, unexpectedly, to behave as Lewis bases.

## 3. Anhydrous Hydrogen Chloride as a Surface Probe

**Figure 3:**
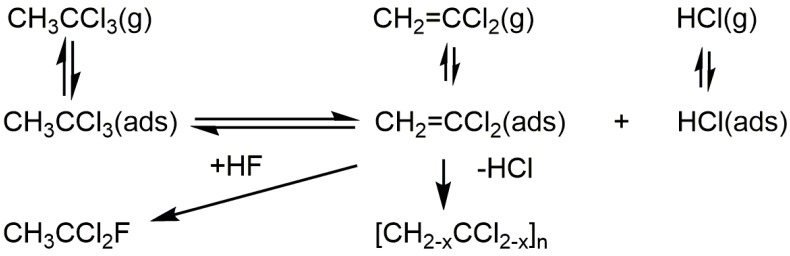
Behavior of CH_3_CCl_3_ on SF_4_-fluorinated γ-alumina. Redrawn from Reference [9].

It is not intuitively obvious that anhydrous HCl might be a useful probe molecule which can be used to compare the surface behavior of different fluorinated surfaces, but this has proven to be the case, particularly for comparisons among nanoscale aluminum fluorides and their conventionally prepared counterparts. The genesis of the approach was a fortuitous observation of reactions that occur when 1,1,1-trichloroethane is exposed to an SF_4_-fluorinated (static conditions, see Table 1) γ-alumina at room temperature. The reactions involved are shown in Figure 3 [9].

The surface-catalyzed reactions shown in Figure 3 are, respectively, a Lewis acid catalyzed dehydrochlorination of CH_3_CCl_3_ and an oligomerization, accompanied by a partial dehydrochlorination, of adsorbed CH_2_=CCl_2_; both have their equivalent reactions using the archetypal Lewis acid, solid aluminum chloride. The presence of retained HF on the surface probably accounts for the fluorination step. This can be made catalytic if the purple, oligomeric supported layer is exposed to a mixture of HF and CH_3_CCl_3_. Although similar behavior is observed when BF_3_ is used to fluorinate γ-alumina (cf. Figure 1), it occurs to a far smaller extent, reflecting, no doubt, the smaller fluorine content of the surface [9].

Dehydrochlorination occurs also when *t*-butyl chloride is exposed to a fluorinated Lewis acidic surface at room temperature, although the chemistry is not as dramatic visually as that derived from CH_3_CCl_3_. It is a useful reaction, however, since Bu*^t^*Cl is readily labeled with [^36^Cl] and the resulting fate of the H^36^Cl produced can be monitored from its radioactivity [12].

The experimental procedures for quantifying β^−^ activity from the long-lived chlorine-36 isotope by Geiger-Müller counting have been well documented in the literature [10,11,12] and are not described here. Although the behavior observed when [^36^Cl]-Bu*^t^*Cl is exposed to SF_4_-fluorinated γ-alumina, β-AlF_3_ and aluminum chlorofluoride (ACF) is similar, there are differences in the details [12]. In particular, the outcomes with SF_4_-fluorinated γ-alumina appear to be dominated by oligomerization reactions that cover the surface, whereas strongly and weakly surface-bound H^36^Cl are observed with ACF. More controversially, a significant fraction of H^36^Cl appears to be retained in the bulk ACF, from which it can be removed by prolonged pumping. The observations using β-AlF_3_ are similar; in this case, however, HCl interacts with residual H_2_O located in the HTB hexagonal channels and is desorbed as H_2_O·HCl. The behaviors of ACF and β-AlF_3_ towards H^36^Cl added directly replicate those described above.

The behavior of nanoscale aluminum(III) fluoride, HS-AlF_3_, towards H^36^Cl parallels that of ACF; weakly and strongly surface-bound H^36^Cl and material retained in the bulk are all observed [13]. Dehydrochlorination of Bu*^t^*Cl is rapid, and HCl is observed immediately in the vapor; the analogous reaction over β-AlF_3_ is far slower. Nanoscale magnesium fluoride, HS-MgF_2_, behaves in an identical way, indicating that unlike rutile MgF_2_, HS-MgF_2_ can behave as a Lewis acid. The composite material, 15 mol % FeF_3_ in HS-MgF_2_, in its behavior towards [^36^Cl]-Bu*^t^*Cl, appears to utilize Lewis acid sites based on Fe^III^ in addition to those involving Mg^II^ [13].

As is evident from the summary given above, the level of detail available about the interactions between HCl and fluorinated surfaces is limited, largely by the corrosive nature of the materials involved. More detailed descriptions become possible for the interactions between anhydrous HCl, which of course has its own corrosion problems, and transitional aluminas such as γ- and η-alumina. FTIR spectroscopy, utilizing temperature-programmed techniques and, more recently, inelastic neutron scattering, has been widely used. Together with a knowledge of the molecular environments of the various surface Lewis acid sites on the aluminas, it is possible to construct realistic hypotheses for events that take place at a chlorinated surface [2,14].

**Figure 4:**
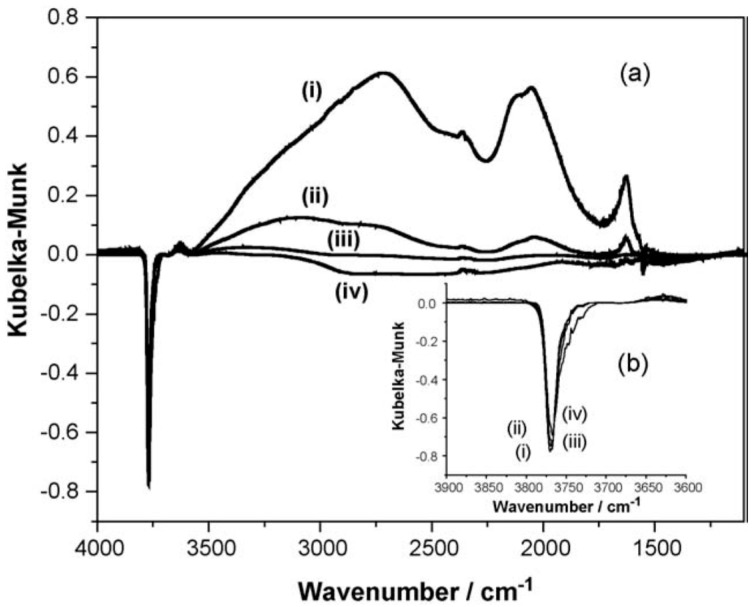
Diffuse reflectance spectra as a function of temperature of a saturated chemisorbed overlayer of HCl: (**a**) 4000–1200 cm^−1^ and (**b**) the free hydroxyl region; (**i**) Saturation spectrum dosed at 293 K. The sample was then progressively warmed to (**ii**) 423; (**iii**) 523; and (**iv**) 623 K. A flow of He was continually passed over the sample while heating to progressively higher temperatures. The sample was held at the desorption temperature for 15 min and then allowed to cool to room temperature, where the spectrum was acquired. All spectra are background subtracted, where the spectrum of the clean activated catalyst has been subtracted from the measured spectrum. Reproduced with permission from Reference [14].

An illustration of what can be achieved under favourable circumstances is the FTIR diffuse reflectance spectra as a function of temperature for a saturated, chemisorbed overlayer of HCl at the surface of η-alumina, as shown in Figure 4 [14].

**Figure 5:**
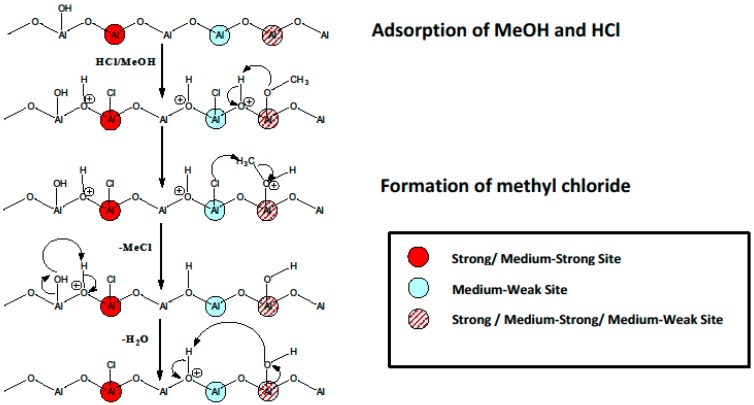
A schematic representation of the site-selective formation of methyl chloride over η-alumina. The red circles represent strong/medium-strong Lewis acid sites; the blue circles represent medium-weak Lewis acid sites; the red/blue shaded circles represent strong/medium-strong/medium-weak Lewis acid sites. The definition and form of these sites are described in D. T. Lundie et al. *J. Phys. Chem. B* **2005**, *109*, 11592–11601 [16]. Reproduced with permission from Reference [15].

For a detailed interpretation of the spectra, the reader is directed to Reference [14]; however, in summary, there is good evidence for weakly adsorbed molecular HCl and dissociatively adsorbed species in which HCl has interacted with Al-O species to form Al-Cl and OH groups, or has replaced a surface Al-OH by Al-Cl with the formation of H_2_O. This type of information is very important in producing realistic sequences of surface events. A very good example is the large-scale selective synthesis of methyl chloride from methanol and HCl, using an η-alumina heterogeneous catalyst [2]. The full sequence of events involving HCl and methanol at the surface is shown schematically in Figure 5 [15].

## 4. Iron(III) and Copper(II) Chlorides as Chlorination Catalysts: Redox and Lewis Acid Possibilities

From a historical standpoint, iron(III) and copper(II) chlorides are two of the most important metal halides in heterogeneous catalysis, being used in a variety of situations, both in laboratory-based studies and in large-scale processes, where catalysis is an important factor.

Anhydrous iron(III) chloride and its hexa-hydrate feature widely in contemporary organic syntheses, since, because of their Lewis acid properties, they can initiate or catalyze a variety of organic transformations [17,18,19,20,21,22,23,24,25,26]. Because of their redox properties arising from the 1e^−^, Fe^III^/Fe^II^ redox transformation, they are also the basis of a useful polymerization process [27,28,29,30,31], for example the polymerization of thiophene. Anhydrous FeCl_3_ has been widely reported as a Lewis acid catalyst for chlorination and hydrochlorination of a variety of hydrocarbons [32,33,34], particularly in large-scale process reactions such as the chlorination of ethene and the hydrochlorination of vinyl chloride [32,33]. Reaction pathways suggested for these types of reactions were traditionally based on polar intermediates but these are probably unrealistic based on current thinking.

Reactions carried out in mild steel that involve Cl_2_ may be problematic, since surface chlorination can occur, leading to a chloride surface that behaves like solid FeCl_3_. As a result, the unwanted chlorination of hydrochlorocarbons can occur; this may be accompanied by oligomerization of unsaturated species [35,36,37]. This type of behavior in the 1,1,2,2-tetrachloroethane/Cl_2_ system was observed in a recent laboratory study of reactions carried out in stainless steel or Pyrex [36,37]. A rather different example of over-chlorination occurred in large-scale reactions of Cl_2_ with CH_2_=CH_2_ and led to unwanted 1,1,2-trichloroethane in the product, 1,2-dichloroethane. The over-chlorination is believed to be catalyzed by the presence of small quantities of FeCl_3_ formed adventitiously, either in solution or at the reactor wall. Considerable effort has been made to inhibit over-chlorination in large-scale processes; for example, by means of FeCl_3_ removal by complexation with various Lewis bases. However, fundamental studies targeted to the identification of molecular species present have apparently never been reported, and in the reports of inhibition by chloride ion–forming tetrachloroferrate(III) anions, differences exist as to the optimum stoichiometry to be used. Because anhydrous FeCl_3_ is extremely hygroscopic, its surface may be complexed by trace water, present even in purified CH_2_ClCH_2_Cl. Molecular species such as FeCl_3_(OH_2_), so formed, are soluble in a hydrochlorocarbon solvent and the resulting solid/solution distribution of chloroferrate(III) species is not simple [38].

The ability of copper(II) chloride, either as a component of a melt or supported on an oxide, to chlorinate a wide variety of saturated and unsaturated hydrocarbons has been known for many years [39,40]; the behavior of pumice-supported CuCl_2_ towards olefins suggests that CuCl_2_ is the chlorinating agent rather than adsorbed Cl* [40]. From a heterogeneous catalytic standpoint, one of the most important uses of CuCl_2_ is as the catalyst for the Deacon reaction, a process for the conversion of HCl to Cl_2_. There is renewed interest in this sequence of reactions, shown in Equations (1)–(4) below, in the context of the utilization of waste HCl. A scheme for the integration of processes producing and using HCl has the aim of “closing the chlorine cycle”; it includes the steps: chlorination, dehydrochlorination and oxychlorination [41].

2Cu^II^Cl_2_ → 2Cu^I^Cl + Cl_2_ (1)

2Cu^I^Cl + 1/2O_2_ → Cu^II^_2_OCl_2_ (2)

Cu^II^_2_OCl_2_ + 2HCl → 2Cu^II^Cl_2_ + H_2_O (3)

Overall

2HCl + 1/2O_2_ → H_2_O + Cl_2_ (4)

Since a Deacon, or oxychlorination, catalyst typically comprises, in addition to CuCl_2_, a support, generally a high-surface-area oxide, and a promoter, such as an ionic halide, for example KCl, Equations (1)–(3) are likely to be an approximation of the surface species present. Furthermore, in a process environment, at the typical operating temperatures used, the species may well be liquid. One of the most detailed studies made to date under laboratory conditions involved CuCl_2_ supported on γ-alumina. The catalyst was used in the oxychlorination of ethene; the product, CH_2_ClCH_2_Cl, underwent dehydrochlorination to CHCl=CH_2_, so the study was very relevant to PVC production [42,43,44,45,46,47,48,49]. Physical methods, particularly surface science–based together with reactor-based experiments, indicate that at least three Cu^II^-containing phases are present and that further mixed Cu^II^/Group I phases can be formed as well.

**Figure 6:**
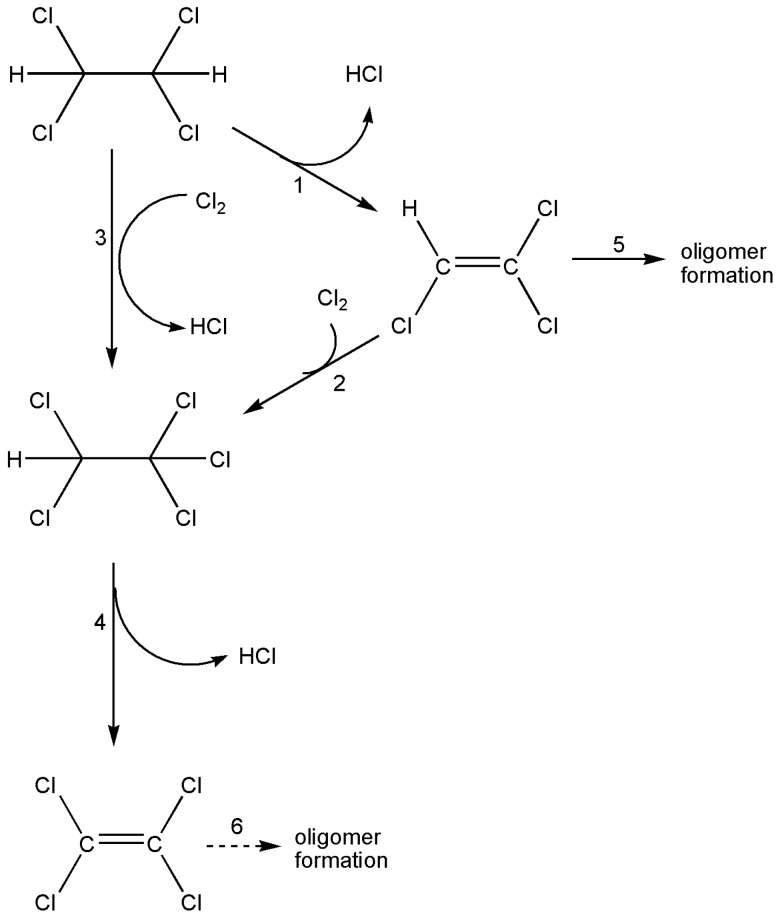
The dehydrochlorination (1, 4) and chlorination (2, 3) processes that connect CHCl_2_CHCl_2_ with CHCl=CCl_2_ and CCl_2_=CCl_2_. Possible oligomer formation (5, 6). Reproduced with permission from Reference [36].

Chlorination or oxychlorination of CH_2_ClCH_2_Cl in the presence of a CuCl_2_/KCl catalyst supported on the clay mineral, attapulgite, leads to a complex mixture of C_2_ chlorohydrocarbons and chlorinated olefins; the products depend on the exact conditions used but careful control can result in CHCl=Cl_2_ or CCl_2_=CCl_2_ being the predominant product. The reaction scheme deduced is shown in Figure 6 [36,37].

There is, inevitably, a trade-off to be made between high conversions and the loss of materials through oligomerization, particularly of CHCl=CCl_2._ Product distributions are the result of competition between chlorination, facilitated by the CuCl_2_ catalyst, and dehydrochlorination. The latter for CHCl_2_CHCl_2_ is initiated by Cl*, whereas dehydrochlorination of CHCl_2_CCl_3_ occurs at Lewis sites on the catalyst.

The Cu^II^ catalyst supports the Deacon reaction to convert the co-product, HCl, to the reactant Cl_2_; however, the reaction is slow compared to the main organo-chlorine transformations.

Phosgene is prepared on a large scale usually by the reaction between carbon monoxide and Cl_2_ over a carbon catalyst. An oxychlorination route has been developed, however, on a laboratory scale using a catalyst comprising CuCl_2_/KCl supported on silica gel [50,51,52]. The challenge in this case is to prepare OCCl_2_ continuously with good conversion and without hydrolysis. This has been achieved by employing a three-stage arrangement corresponding to Equations (1)–(4), viz. Equations (5)–(8).

2CuCl_2_ + CO → 2CuCl + COCl_2_ (5)

2CuCl + 1/2O_2_ → Cu_2_OCl_2_ (6)

Cu_2_OCl_2_ + 2HCl → 2CuCl_2_ + H_2_O (7)

Overall:

CO + 1/2O_2_ + 2HCl → COCl_2_ + H_2_O (8)

## 5. Conclusions

The two parts of this short review have different objectives. In the first part, we sought to show that selected metal fluorides prepared in conventional and unconventional ways have many similarities in their Lewis acidity, using a variety of probe molecules, including the very weak Lewis base anhydrous HCl. In the second part, some aspects of FeCl_3_ and, particularly, CuCl_2_, chemistry demonstrated the complexity in their catalytic behavior. The challenge for those who are synthesizing nanoscale halides is to investigate whether there is a parallel chemistry to be uncovered.

## Figures and Tables

**Table 1:** Some properties of fluorinated γ-alumina [8].

Fluorination Conditions ^a^	BET Area (m^2^·g^−1^)	Fluorine Content (%)	Other Properties
SF_4_ static conditions, nominally room temperature, 2 h, procedure repeated ×2	80–90 ^b^	ca. 22 ^c^	Some Brønsted sites also
SF_4_ 20% in N_2_, flow conditions, 523 K, 2 h	67	47.1	γ-alumina present; possibly an amorphous phase also
CHF_3_ 20% in N_2_, then 100%, flow conditions, 623 K, total time 5 h	34 ^d^	58.4	α- and β-AlF_3_ present; possibly an amorphous phase

^a^ In all cases samples were calcined before use; ^b^ Before fluorination 110 m^2^·g^−1^; ^c^ Calculated from a radiotracer study; ^d^ Before fluorination 240 m^2^·g^−1^.

## References

[1] Tressaud A. (2010). Functionalised Inorganic Fluorides.

[2] Lennon D., Winfield J.M., Parvulescu V.I., Kemnitz E. (2016). Heterogeneous Catalysts used for Large-Scale Syntheses of Selected Catalytic Applications.

[3] Le Bail A., Jacaboni C., Leblanc M., De Pape R., Duroy H., Fourquet J.L. (1988). Crystal Structure of the Metastable form of Aluminium Trifluoride βAlF_3_ and the Gallium and Indium Homologues. J. Solid State Chem..

[4] Hess A., Kemnitz E. (1994). Characterisation of Catalytically Active Sites on Aluminium Oxides, Hydroxyfluorides and Fluorides in Correlation with their Catalytic Behaviour. J. Catal..

[5] Kemnitz E., Winfield J.M., Nakajima T., Žemva B., Tressaud A. (2000). Fluoride Catalysts: Their Application to Heterogeneous Catalytic Fluorination and Related Processes. Advanced Inorganic Fluorides: Synthesis, Characterization and Applications.

[6] Krespan C.G., Petrov V.A. (1996). The Chemistry of Highly Fluorinated Carbocations. Chem. Rev..

[7] Krahl T., Kemnitz E. (2006). The Very Strong Lewis Acids Aluminium Chlorofluoride (ACF) and Bromofluoride (ABF)—Synthesis, Structure and Lewis Acidity. J. Fluor. Chem..

[8] Kemnitz E., Skapin T., Winfield J.M., Roesky H.W. (2013). Preparation of Fluorinated γ-Alumina. Efficient Preparations of Fluorine Compounds.

[9] Klapötke T.M., McMonagle F., Spence R.R., Winfield J.M. (2006). γ-Alumina-Supported Boron Trifluoride: Catalysis, Radiotracer Studies and Computations. J. Fluor. Chem..

[10] Vimont A., Daturi M., Winfield J.M., Tressaud A. (2010). Investigation of Surface Acidity using a Range of Probe Molecules. Functionalised Inorganic Fluorides.

[11] Winfield J.M. (2009). Investigating Acidity of Metal Fluoride Surfaces by Spectroscopic and Chemical Methods. J. Fluor. Chem..

[12] Nickkho-Amiry M., Winfield J.M. (2007). Investigation of Fluorinated Surfaces by means of Radio-Labelled Probe Molecules. J. Fluor. Chem..

[13] Nickkho-Amiry M., Eltanany G., Wüttke S., Rüdiger S., Kemnitz E., Winfield J.M. (2008). A Comparative Study of Surface Activity in the Amorphous, High Surface Area Solids, Aluminium Fluoride, Magnesium fluoride and Magnesium Fluoride containing Iron(III) or Aluminium(III) Fluoride. J. Fluor. Chem..

[14] McInroy A.R., Lundie D.T., Winfield J.M., Dudman C.C., Jones P., Parker S.F., Lennon D. (2006). The Interaction of Alumina with HCl: An Infrared Spectroscopy, Temperature-Programmed Desorption and Inelastic Neutron Scattering Study. Catal. Today.

[15] McInroy A.L., Winfield J.M., Dudman C.C., Jones P., Lennon D. (2016). The Development of a New Generation of Methyl Chloride Synthesis Catalysts. Faraday Discuss..

[16] Lundie D.T., McInroy A.R., Marshall R., Winfield J.M., Jones P., Dudman C.C., Parker S.F., Mitchell C., Lennon D. (2005). Improved Description of the Surface Acidity of η-Alumina. J. Phys. Chem. B.

[17] Zhang X., Fan X., Niu H., Wang J. (2003). An Ionic Liquid As a Recyclable Medium for the Green Preparation of α,α′-Bis(substituted benzylidine)cycloalkanones Catalysed by FeCl_3_·6H_2_O. Green Chem..

[18] Kabalka G.W., Yao M.-L., Borella S., Goins L.K. (2007). Iron Trichloride Mediated Allylation of Lithium Alkoxides through an Unusual Carbon-Oxygen Bond Cleavage. Organometallics.

[19] Xa W.-W., Lin M., Huang Y., Chen L., Zhan Z.-P. (2009). Iron Trichloride Catalysed One-Pot Synthesis of Substituted Oxazoles from Propargylic Acetates and Amides under Microwave Irradiation. Lett. Org. Chem..

[20] Cantagrei G., De Canné-Carnavalet B., Meyer C., Cossy J. (2009). IronTrichloride Promoted Cyclisation of *O*-Alkynylaryl Isocyanates. Synthesis of 3-(Chloromethylene)Oxindoles. Org. Lett..

[21] Schröder K., Enthaler S., Bitterlich B., Schulz T., Spannenberg A., Tse M.K., Junge K., Beller M. (2009). Design of and Mechanistic Studies on a Biomimetic Iron-Imidazole Catalyst System for Epoxidation of Olefins with Hydrogen Peroxide. Chem. Eur. J..

[22] Buchwald S.L., Bolm C. (2009). On the Role of Metal Contaminants in Catalyses with FeCl_3_. Angew. Chem. Int. Ed..

[23] Schröder K., Junge K., Spannenberg A., Beller M. (2010). Design of a Bio-Inspired Imidazole-Based Iron Catalyst for Epoxidation of Olefins: Mechanistic Insights. Catal. Today.

[24] Su L., Lei C.-Y., Fan W.-Y., Liu L.-X. (2011). FeCl_3_-Mediated Reaction of Alkynols with Iodine: An Efficient and Convenient Route to Vinyl Iodides. Synth. Commun..

[25] Blevins D.W., Yao M.-L., Yong L. (2011). Iron Trichloride Promoted Hydrolysis of Potassium Organotrifluoroborates. Tetrahedron Lett..

[26] Yeh M.-C.P., Fang C.-W., Lin H.-H. (2012). Facile Synthesis of Azaspirocycles via Iron Trichloride Promoted Cyclisation/Chlorination of Cyclic 8-Aryl-5-aza-5-tosyl-2-en-7-yn-1-ols. Org. Lett..

[27] Della Casa C., Bertinelli F., Costa Bizzarri P., Salatelli E. (1995). Properties of a Hydroxydecyl-Functionalized Polythiophene Synthesized by the Iron Trichloride Route. Adv. Mater..

[28] Tanaka K., Mihara T., Koide N. (2004). Synthesis and Physical Properties of Regioregular Poly(3-Alkoxy-4-Methylthiophene)s. Polym. J..

[29] Svoboda J., Bláha M., Sedláček J., Vohlídal H., Balcar H., Mav-Golež I., Žigon M. (2006). New Approaches to the Synthesis of Pure Conjugated Polymers. Acta Chim. Slov..

[30] Cloutet E., Mumtaz M., Cramail H. (2009). Synthesis of PEDOT Latexes by Dispersion Polymerization in Aqueous Media. Mater. Sci. Eng. C.

[31] Lanzi M., Paganin L., Errani F. (2012). Synthesis, Characterization and Photovoltaic Properties of a New Thiophene-Based Double-Cable Polymer with Pendent Fullerene Group. Polymer.

[32] Reed D.J., Kroschwitz J.I., Howe-Grant M. (1993). Chlorocarbons and Chlorohydrocarbons. Kirk-Othmer Encyclopaedia of Chemical Technology.

[33] Weissermel K., Arpe H.-J. (2003). Industrial Organic Chemistry.

[34] Delaude L., Lazlo P. (1990). Aromatic Chlorination of Toluene and of Anisole Using Clay-Supported FeCl_3_ and *m*-Chloroperbenzoic Acid. A Biomimetic Approach. Catal. Lett..

[35] Kawaguchi T., Suzuki Y., Saito R. (1970). Trichloroethylene and Perchloroethylene Coproduction by Ethylene Process. Ind. Eng. Chem..

[36] Sutherland I.W., Hamilton N.G., Dudman C.C., Jones P., Lennon D., Winfield J.M. (2011). Chlorination and Dehydrochlorination Reactions Relevant to the Manufacture of Trichloroethene and Tetrachloroethene. Part 1. Reaction Pathways. Appl. Catal. A Gen..

[37] Sutherland I.W., Hamilton N.G., Dudman C.C., Jones P., Lennon D., Winfield J.M. (2014). Chlorination Reactions Relevant to the Manufacture of Trichloroethene and Tetrachloroethene. Part 2. Effects of Chlorine Supply. Appl. Catal. A Gen..

[38] Taylor D.S.C. (2002). Catalysis in the System 1,2-Dichloroethane, Iron(III) Chloride. Ph.D. Thesis.

[39] Fontana C.M., Gorin E., Kidder G.A., Meredith C.S. (1952). Ternary System, Cuprous Chloride-Cupric Chloride-Potassium Chloride and its Equilibrium Chlorine Pressures. Ind. Eng. Chem..

[40] Arganbright R.P., Yates W.F. (1962). Chlorinations with Cupric Chloride. J. Org. Chem..

[41] Cavani F. (2010). Catalytic Selective Oxidation: The Forefront in the Challenge for a more Sustainable Chemical Industry. Catal. Today.

[42] Leofanti G., Padovan M., Garilli M., Carmello D., Zecchina A., Spoto G., Bordiga S., Turnes Palomino G., Lamberti C. (2000). Alumina-Supported Copper Chloride 1. Characterization of freshly Prepared Catalyst. J. Catal..

[43] Leofanti G., Padovan M., Garilli M., Carmello D., Marra G.L., Zecchina A., Spoto G., Bordiga S., Lamberti C. (2000). Alumina-Supported Copper Chloride 2. Effect of Aging and Thermal Treatments. J. Catal..

[44] Leofanti G., Marsella A., Cremaschi B., Garilli M., Zecchina A., Spoto G., Bordiga S., Fisicaro P., Berlier G., Prestipino C. (2001). Alumina-Supported Copper Chloride 3. Effect of Exposure to Ethylene. J. Catal..

[45] Leofanti G., Marsella A., Cremaschi B., Garilli M., Zecchina A., Spoto G., Bordiga S., Fisicaro P., Prestipino C., Villain F. (2002). Alumina-Supported Copper Chloride 4. Effect of Exposure to O_2_ and HCl. J. Catal..

[46] Lamberti C., Prestipino C., Bonino F., Capello L., Bordiga S., Spoto G., Zecchina A., Diaz Moreno S., Cremaschi B., Garilli M. (2002). The Chemistry of the Oxychlorination Catalyst: An In Situ, Time-Resolved XANES Study. Angew. Chem. Int. Ed..

[47] Prestipino C., Bordiga S., Lamberti C., Vidotto S., Garilli M., Cremaschi B., Marsella A., Leofanti G., Fisicaro P., Spoto G. (2003). Structural Determination of Copper Species on the Alumina-Supported Copper Chloride Catalyst: A Detailed EXAFS Study. J. Phys. Chem. B.

[48] Muddada N.B., Olsbye U., Caccialupi L., Cavani F., Leofanti G., Gianolio D., Bordiga S., Lamberti C. (2010). Influence of Additives in Defining the Active Phase of the Ethylene Oxychlorination Catalyst. Phys. Chem. Chem. Phys..

[49] Muddada N.B., Olsbye U., Leofanti G., Gianolio D., Bonino F., Bordiga S., Fuglerud T., Vidotto S., Marsella A., Lamberti C. (2010). Quantification of Copper Phases, their Reducibility and Dispersion in Doped-CuCl_2_/Al_2_O_3_ Catalysts for Ethylene Oxychlorination. Dalton Trans..

[50] Zhang T., Troll C., Rieger B., Kintrup J., Schlüter O.F.-K., Weber R. (2009). Reaction Kinetics of Oxychlorination of Carbon Monoxide to Phosgene based on Copper(II) Chloride. Appl. Catal. A Gen..

[51] Zhang T., Troll C., Rieger B., Kintrup J., Schlüter O.F.-K., Weber R. (2009). Composition Optimisation of Silica-Supported Copper(II) Chloride Substance for Phosgene Production. Appl. Catal. A Gen..

[52] Zhang T., Troll C., Rieger B., Kintrup J., Schlüter O.F.-K., Weber R. (2010). Oxychlorination of CO to Phosgene in a Three-Step Reaction Cycle and Corresponding Catalytic Mechanism. J. Catal..

